# Impacts of Long-Term Micronutrient Fertilizer Application on Soil Properties and Micronutrient Availability

**DOI:** 10.3390/ijerph192316358

**Published:** 2022-12-06

**Authors:** Shuzhuan Wang, Lei Xu, Mingde Hao

**Affiliations:** 1 Key Laboratory of Natural Disaster and Remote Sensing of Henan Province, Nanyang Normal University, Nanyang 473061, China; 2 Institute of Soil and Water Conservation, Chinese Academy of Sciences and Ministry of Water Resources, Yangling, Xianyang 712100, China

**Keywords:** micronutrient availability, soil properties, micronutrient fertilizer, Loess Plateau

## Abstract

Deficiencies of micronutrients in calcareous soils have been reported in different areas of China’s Loess Plateau. The objective of this research was to study the influence of the continuous application of micronutrient fertilizers on soil properties and micronutrient availability in this region. The micronutrient fertilizer field plot experiment began in 1984. It included Zn, Mn and Cu fertilizer treatments and the control treatment. The crop system was continuously cropped winter wheat. The soil properties and available Zn, Mn, Cu and Fe were measured. Their relationships were analyzed through correlation and path analysis. After 31 years, the soil pH, CaCO_3_ and available P levels decreased; in contrast, the organic matter, fulvic acid, reducing substances and soil moisture levels in the surface soil increased in the micronutrient fertilized treatments compared to the control treatment. Cu and Zn fertilizers promoted the available Cu and Zn levels in the surface and deep soil, but available Mn was not significantly affected by Mn fertilizer. It can be seen from the interaction between the micronutrient availability and micronutrient fertilizers that Zn, Cu and Mn fertilizers can increase the available Fe level; Mn fertilizer can increase the available Cu level, and Cu and Mn fertilizers can increase the available Zn level. This means that Fe, Cu and Zn availability were easy to implement, whereas the soil-available Mn was difficult to improve in calcareous soils on the Loess Plateau. Fulvic acid and organic matter showed a significant and direct effect on the available Zn; the available Mn and Fe were mainly affected by the soil CaCO_3_ and moisture; the available Cu was mainly affected by the soil organic matter, available P and reducing substances. These results indicate the importance of organic matter in calcareous soils; it can not only directly affect the availability of micronutrients but also indirectly affect their availability through the indirect interaction with fulvic acid, reducing substances, available P and CaCO_3_. The above conclusions suggest that the long-term micronutrient fertilizers changed some important soil properties and increased the micronutrient availability in the loess-derived soil.

## 1. Introduction

Micronutrients, such as Zn, Fe, Mn, Cu and so on, are indispensable for life. When the concentrations of micronutrients in soil are excessively low, their supply is insufficient to meet the needs of organisms. Conversely, when their concentrations in soil are excessively high, they can have toxic effects. According to Aubert and Pinta, the total content of micronutrients, such as Mn and Fe, in most soils is high; however, Mn and Fe deficiency in soils has occurred worldwide [1]. Today, more than 50% of soil lacks micronutrients, which directly leads to a reduction in the crop yield and quality of agricultural products. More than 2 billion people in the world are in a state of malnutrition, and the lack of micronutrients has had an adverse impact on human health [2,3,4]. This deficiency is mainly caused by the availability of micronutrients, which cannot provide a sufficient amount necessary for plant uptake. Thus, research on the availability of micronutrients in soil has become a major focus in the fields of agriculture and environmental science [5,6].

Heilu soil, which corresponds to a Calcarid Regosol according to the FAO/UNESCO classification system (1988), is a typical soil type on the Loess Plateau. Heilu soil has good permeability because it is derived from loess and contains more than 50% silt and 7–15% calcareousness [7]. This property is not beneficial for nutrient accumulation. Studies conducted in the United States, Canada, the United Kingdom, Australia, India and China have shown that calcareous soil is often deficient in micronutrients, especially on the Loess Plateau, because soil erosion is a major problem in this region [8,9,10,11,12]. Yu et al. showed that the average total Cu concentration in soils on the Loess Plateau was 22.5 mg kg^−1^, which was slightly higher than the average values in the soils in China (22 mg kg^−1^) and worldwide (20 mg kg^−1^). The average total Mn concentration in the soils on the Loess Plateau was 537 mg kg^−1^, which was lower than the average values in the soils in China (710 mg kg^−1^) and worldwide (850 mg kg^−1^). The average total Zn concentration in the soils on the Loess Plateau was 69.1 mg kg^−1^, which was lower than the average value in the soils in China (100 mg kg^−1^) and higher than the average value worldwide (50 mg kg^−1^). Compared with the critical value, the average concentration of available Cu, Mn, Zn and Fe was 0.93 (>0.5), 7.7 (>7.0), 0.51 (>0.5) and 5.6 mg kg^−1^ (>2.5 mg kg^−1^), respectively [13]. Approximately 20.8, 48.3, 56 and 11.2% of the land area was low in Cu, Mn, Zn and Fe, respectively. With the development of modern agriculture, the absorption and utilization of Cu, Mn, Zn and Fe in soil by crops is in constant flux. Therefore, the deficiency of micronutrients has largely limited the agricultural production on the Loess Plateau, and the underlying mechanisms should be further studied.

The availability of micronutrients in soils for plant uptake are affected not only by natural factors such as soil properties but also by human factors such as agricultural practices [13,14,15]. The existing research mainly focuses on organic matter, available P, CaCO_3_ and pH in order to explore the mechanism as much as possible, and we determined the content of humic acid, fulvic acid and the total amount of reducing substances. Mn, Cu and Fe are all valence-variable elements, and they can interact in soil. For example, as a very important metallic redox catalyst, Mn can govern the behavior of other micronutrients in soils [6]. Therefore, the long-term application of micronutrient fertilizers can have different effects, such as promotion, antagonism, etc., on the micronutrient availability in soil [16,17,18]. Different from the previous studies, this study analyzed the effects of different kinds of micronutrient fertilizers on micronutrient availability through long-term micronutrient fertilizer experiments, which can help us to understand the interaction among micronutrients; thus, this study can protect and make use of any potential available pool to satisfy plant needs and maintain agricultural sustainability.

Here, the available fraction and soil properties were investigated in semiarid soils under the long-term (31-year) application of micronutrient fertilizers on the Loess Plateau. The objectives were to (i) analyze the changes in micronutrient availability and soil properties under the 31-year application of micronutrient fertilizers and (ii) explore the relationships between micronutrient availability and soil properties. This study could provide suggestions for improving micronutrient availability through agricultural practices. It could also provide insights into how micronutrient fertilizers could be optimally applied in semiarid soils and in other areas with similar features.

## 2. Materials and Methods

### 2.1. Study Area

The study area is located in the dry land of Shilipu Village, Changwu County, Shaanxi Province in the south-central region of the Loess Plateau, China (35°12′ N, 107°40′ E). The experiment was initiated in 1984 and was designed by Peng Lin and Li Yushan, based on the results of their research [19]. The soil in the study area is a Heilu soil developed from deep, thick, medium loam Malan loess, which corresponds to a Calcarid Regosol according to the FAO/UNESCO classification system (1988). It is loose and uniform with good permeability and medium fertility. When the experiment was initiated in the autumn of 1984, the organic matter content was 10.4 g kg^−1^, available P was 3.0 mg kg^−1^, CaCO_3_ was 108.4 g kg^−1^, pH was 8.3, total nitrogen was 0.60 g kg^−1^, available nitrogen was 37.00 mg kg^−1^, total phosphorus was 0.659 g kg^−1^, and available potassium was 129 mg kg^−1^ in cultivated soil. The altitude of the study area is 1200 m, the average annual temperature is 9.1 °C, and the active accumulated temperatures of ≥0 and 10 °C are 3866 and 3029 °C, respectively. The frost-free period is 171 days. The study area experiences a warm, temperate, semihumid, continental monsoon climate, and crops are harvested once a year. The soil fertility and geomorphic characteristics of the test area are typical for the Loess Plateau.

### 2.2. Experimental Design

Continuously cropped winter wheat (*Triticum aestivum* L.) was planted in the experiment. The wheat varieties selected were Qinmai #4 from 1984 to 1985, Changwu #131 from 1986 to 1995 and Changwu #134 from 1996 to 2015. Wheat was sown in mid-September and harvested by June in the next year, then land was kept fallow from June to September each year. The experiment was set up in a randomized block design with 3 replicates: CK application (N 60 kg hm^−2^ and P 60 kg hm^−2^), Cu application (N 60 kg hm^−2^, P 60 kg hm^−2^ and CuSO_4_ 15 kg hm^−2^ containing 25.45% Cu), Mn application (N 60 kg hm^−2^, P 60 kg hm^−2^ and MnSO_4_.H_2_O 22.5 kg hm^−2^ containing 25.80% Mn) and Zn application (N 60 kg hm^−2^, P 60 kg hm^−2^ and ZnSO_4_.7H_2_O 15 kg hm^−2^ containing 22.73% Zn). Prior to seeding, N fertilizer in the form of urea and P fertilizer as super-phosphate were spread onto the plots and then incorporated to a depth up to 15 cm. Wheat seeds and micronutrient fertilizers were placed on the sides of the furrows up to 10 cm deep, which were made with a small, V-shaped plow, then a roller was pulled over the soil to cover the seeds and micronutrient fertilizers. The plot size was 5.5 × 4 m. Agricultural management of the experimental plots mimicked that of field crop management practices.

### 2.3. Sample Collection

Soil samples were collected in September 2015. Three random cores were taken from each plot to form a composite sample. The soil sampling depth was 60 cm, and the sampling interval was 15 cm. A total of 48 soil samples were collected. All soil samples were air-dried, ground and passed through 1 mm and 0.25 mm nylon screens.

### 2.4. Chemical Analysis

According to the method described by Page et al., soil pH was analyzed at a soil-to-water ratio of 1:2 with a glass electrode, and soil-available P was analyzed by extracting 5 g soil (<1.0 mm) with 100 mL of 0.5 mol L^−1^ NaHCO_3_ and 50 mL of 1 mol L^−1^ NH_4_OAc [20]. Soil organic matter was tested using titration developed by Walkley and Black [21]. Soil CaCO_3_ was tested using manometry proposed by Agrochemistry Commission and Soil Science Society of China. Soil moisture was tested using drying method. The total amount of reducing substances was extracted with aluminum sulfate and titrated with potassium dichromate. Humic acid and fulvic acid were determined using the extraction method of sodium pyrophosphate and sodium hydroxide mixture according to the technical specification for soil analysis published by China Agricultural Press.

According to Lindsay and Norvell, the available fraction of micronutrients in soil was tested using the diethylene thiamine pentacetic acid (DTPA) extraction procedure which was suitable for the calcareous soils [22]. First, 10 g of air-dried soil (<1.0 mm) was extracted with 20 mL of 0.005 mol L^−1^ DTPA, 0.01 mol L^−1^ CaCl_2_ (pH = 7.3) and 0.1 mol L^−1^ triethanolamine. After shaking for 2 h at 25 °C, the suspension was filtered through Whatman No. 5 filter paper and then was transferred into a polyethylene bottle at 4 °C for analysis.

The contents of soil micronutrient fractions were measured using atomic absorption spectrometry after extraction.

### 2.5. Quality Control

All laboratory glassware for micronutrient analysis was presoaked in 50 mg L^−1^ detergent solution for no less than 8 h and washed using tap water, then soaked in 150 mL L^−1^ 14% (*v*/*v*) HNO_3_ solution overnight and rinsed using deionized water before use. Reagents used in this study were of analytical grade. Standard soil reference materials (GBW07408) from the Institute of Geophysical and Geochemical Exploration, Chinese Academy of Geological Sciences, were carried through the digestion and analysis procedure as a part of the assurance-quality control protocol.

### 2.6. Statistical Analysis

The data were subjected to one-way analysis of variance (ANOVA) using the SPSS software package (version 15.0) (Statistical Graphics Corp., Princeton, NJ, USA). Means and standard errors were calculated for three replicates. Means were compared using Duncan’s multiple range test at a significance level of 0.05.

SPSS software was also used to perform correlation and path analyses. Path analysis was improved on the basis of multiple regression analysis. Path analysis decomposes correlation coefficients into direct path coefficients (the direct effect of an independent variable on the dependent variable) and indirect path coefficients (the indirect effect of the independent variable on the dependent variable through other independent variables).

Assuming that Y and X_1_, X_2_……, X_n_ have linear relation, the regression equation can be written as follows:

Y = B_0_ + B_1_X_1_ + B_2_X_2_ + … + B_n_X_n_.

The steps of path analysis for Y and X_1_, X_2_……, Xn are given below:

(1) Coefficients of correlation R_xiy_, R_xixj_ (i, j = 1, 2, …, n; i < j) are obtained from the correlation coefficient and test output results in the regression analysis.
(2) Direct path coefficient (P_iy_) is the standardized coefficients beta in the regression analysis.
(3) The indirect path coefficient (R_iy_) of X_i_ to Y through X_j_ is:
  R_iy_ = R_xixj_ × P_jy_
(4) Any coefficient of correlation can be considered as an algebraic sum of direct effects and indirect effects of relevant characters under investigation. That is:

R_1y_ = P_1y_ + R_12_ + R_13_ + …… + R_1j_

R_2y_ = P_2y_ + R_21_ + R_23_ + …… + R_2j_

……

R_iy_ = P_iy_ + R_i1_ + R_i3_ + …… + R_ij_

## 3. Results and Discussion

### 3.1. Soil Properties

#### 3.1.1. Soil pH

Compared with the control, the soil pH value increased with the long-term use of micronutrient fertilizers; this might be caused by the metal ions contained in the micronutrient fertilizers (Table 1). The soil pH value of each treatment increased gradually with the increase in soil depth. The pH value of the topsoil with Cu fertilizer was the lowest (8.23), followed by Zn fertilizer and Mn fertilizer. The pH value of the topsoil with Mn fertilizer was the highest, which increased by 0.2 pH units compared with the control. The long-term application of the fertilizers always led to a decrease in soil pH [5]. This might be due to the release of all kinds of acids and the nitrification of NH_4_^+^. The uptake of N as NH_4_^+^ by the crops might also contribute to the decrease in soil pH. Conyers et al. found a 0.4 pH unit reduction in the topsoil under the application of urea [23]. Wei et al. reported a reduction in pH which ranged from 0.03 to 0.27 units [5]. There was little difference on the soil pH with micronutrient fertilizers.

#### 3.1.2. Soil Organic Matter

The application of micronutrient fertilizers increased the soil organic matter in the plough layer, and its content in each treatment decreased gradually with the increase in soil depth (Table 1). The higher content was with the application of Cu fertilizer, which increased by 25% compared with the control, followed by the application of the Mn fertilizer which increased by 17%, and the increase with the Zn fertilizer was the lowest. Wei et al. reported 103% higher results in continuous clover and 61% higher results with N, P and organic fertilizer treatments in crop-legume rotation [5]. This indicated that crop rotation systems, legume-based cropping systems, and N, P and organic fertilizer treatments showed a significant difference with soil organic matter compared to micronutrient fertilizers.

#### 3.1.3. Soil CaCO_3_

Soil CaCO_3_ mostly decreased after the long-term use of micronutrient fertilizers, and its content in each treatment first increased and then decreased with the increase in soil depth, which might be due to the leaching and deposition of CaCO_3_ of the surface soil (Table 1). Compared with the control, the most significant decrease was with the application of Cu fertilizer; the content of CaCO_3_ in 0–15, 15–30, 30–45 and 45–60 cm of soil decreased, respectively, by 32.35, 41.25, 35 and 30.68%. Soil CaCO_3_ decreased after the long-term use of micronutrient fertilizers. This might be due to the release of all kinds of acids, which increased the solubility of calcium in the soil, or it was due to the deposition of calcium carbonate to the lower layer.

#### 3.1.4. Soil-Available P

Soil-available P mostly decreased after long-term micronutrient fertilizer application compared with the control, and its content in each treatment decreased gradually with the increase in soil depth (Table 1). The largest decrease in the arable soil was with the application of Zn fertilizer, followed by Mn fertilizer, which decreased, respectively, by 55 and 50% compared with the control, and it decreased the least with the Cu fertilizer (17%). P was removed from the soil by plant uptake and harvest, or it might be related to the interaction such as inhibition and antagonism among P, Cu, Zn and Mn [7,12].

#### 3.1.5. Soil Humic Acid

After the long-term application of micronutrient fertilizers, the soil humic acid mostly decreased, and its contents varied with the increase in soil depth (Table 1). The highest decrease in the surface soil was under the application of the Mn fertilizer, which decreased by 77.87%, followed by 73.77% under the Cu fertilizer and 65.16% under the Zn fertilizer. Humic acid can complex metal ions and form aqueous complexes with micronutrients, though not to the same extent as many synthetic chelating agents [24]; however, there were few studies to determine humic acid under long-term micronutrient fertilizers.

#### 3.1.6. Soil Fulvic Acid

Compared with the control, the application of Cu fertilizer in the topsoil and 30–60 cm of soil increased the content of fulvic acid (16.97%) which decreased under the application of the Mn fertilizer and Zn fertilizer (Table 1). The largest increase in fulvic acid in the subsurface soil was with the application of the Zn fertilizer, which increased by 2.27 times. The 30–45 and 45–60 cm of soil decreased, respectively, by 52.23 and 80.78% under the application of the copper fertilizer. The ratio of humic acid to fulvic acid was less than 1. Unlike humic acid, fulvic acid has a different molecular structure; it can also absorb metal ions [24] or occur in complex reactions; however, there were also few studies to determine fulvic acid under long-term micronutrient fertilizers.

#### 3.1.7. Soil Total Amount of Reducing Substances

After 31 years of micronutrient fertilizer application, the total amount of reducing substances in the surface soil increased, indicating that micronutrient fertilizers were conducive to the increase in the total amount of reducing substances in the topsoil (Table 1). With the application of the Cu fertilizer in the topsoil, it increased the most, with an increase of 24.74%. The total amount of reducing substances decreased gradually with the increase in soil depth in each treatment. The redox reaction is an important mechanism of soil micronutrient transformation, which is most important for iron and manganese. There were few studies to determine the soil total amount of reducing substances under long-term micronutrient fertilizers.

#### 3.1.8. Soil Moisture

Compared with the control, soil moisture under the long-term use of micronutrient fertilizers basically increased; it increased the most with the application of the Mn fertilizer (Table 1). The soil of 0–15, 15–30, 30–45 and 45–60 cm increased by 18.86, 9.38, 10.26 and 4.02%, respectively. The soil moisture content of each treatment increased gradually with the increase in soil depth. Soil moisture is an important limiting factor for rainfed farmlands on the Loess Plateau. Therefore, it is necessary to determine soil moisture under long-term micronutrient fertilizers.

### 3.2. Effects of Long-Term Application of Micronutrient Fertilizers on Micronutrient Availability

#### 3.2.1. Available Cu

**Figure 1:**
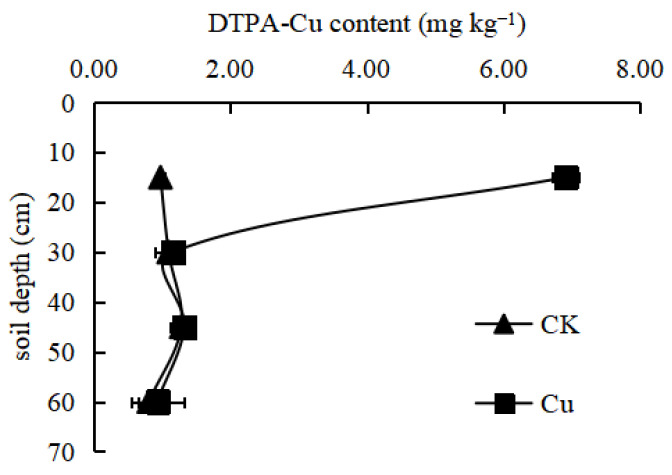

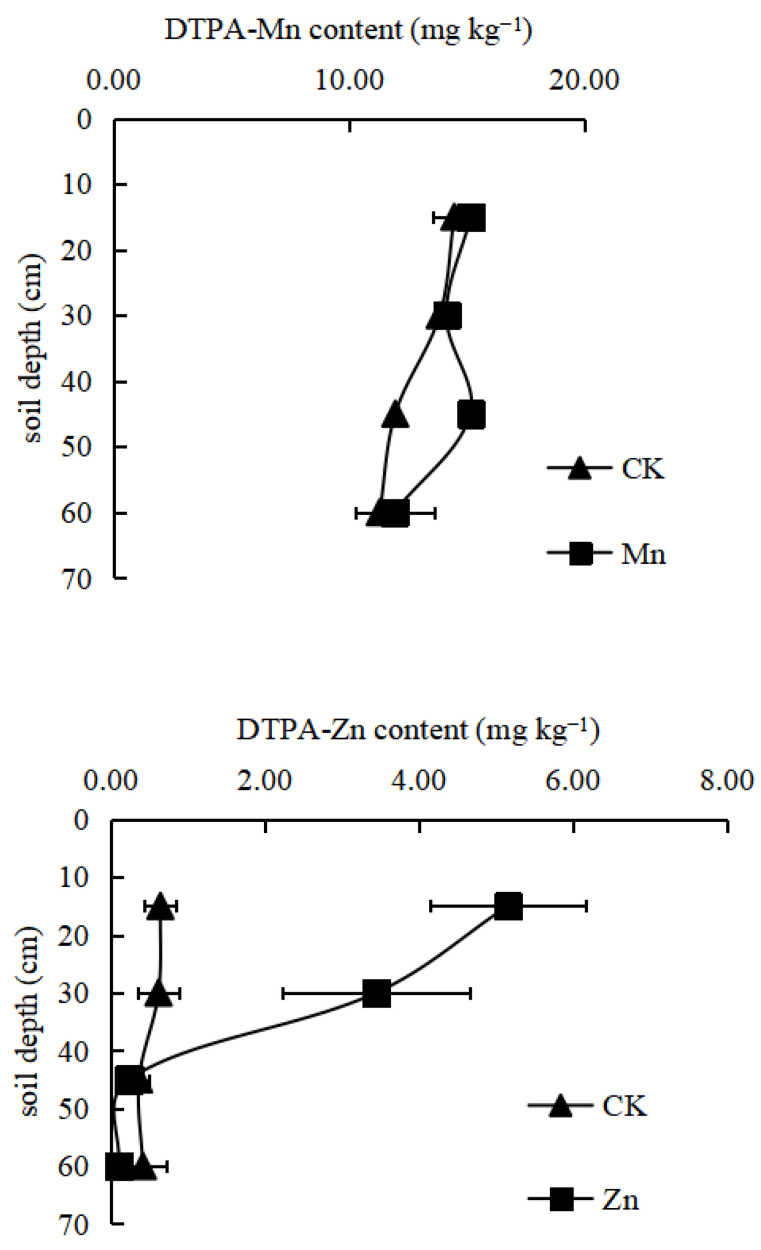
Effects of the long-term application of micronutrient fertilizers on micronutrient availability. Values are means with standard deviations shown by the vertical bars (n = 3).

In the absence of Cu application (Figure 1), the available Cu in soil gradually decreased with the soil depth, and it ranged from 0.95 to 1.29 mg kg^−1^. Under Cu application, the available Cu was significantly higher than in the control. The available Cu in the surface soil increased 6.03-fold compared with the control, and the available Cu was maintained between 6 and 7 mg kg^−1^. Another enrichment of Cu was most pronounced in the 30 cm soil layer. Cu easily precipitates with carbonates and bicarbonates in calcareous soil; in addition, Cu easily forms complexes with organic matter, which can impede its specific adsorption. Therefore, Cu does not easily migrate in soils and can easily become enriched, which affects its availability [12]. The distribution of the available Cu in this study could reflect the above chemical characteristics of Cu.

#### 3.2.2. Available Mn

In the absence of Mn application, the available Mn gradually decreased with the soil depth (Figure 1). Mn application increased the soil-available Mn, although the increases were small. Wilson et al. also obtained the same conclusion [25]. The largest increase (26.54%) was in the 30–45 cm soil depth. The increases in the other soil depths were less than 6%. Overall, the effect of Mn fertilizer on the increase in available Mn was weak. This might stem from the fact that Mn can easily bind to oxides for fixation and can be easily oxidized. The oxidation capacity is weaker in deep-layer soils, which is not conducive to the leaching of low-valence Mn ions. Consequently, Mn application increased the available Mn in the deep-layer soils.

#### 3.2.3. Available Zn

In the absence of Zn application, the soil-available Zn gradually decreased with the soil depth (Figure 1). Application of the Zn fertilizer increased the available Zn in the 0–30 cm soil layer. The available Zn in the surface soil and subsurface soil were increased 6.97- and 4.54-fold, respectively. The available Zn in the 30–45 and 45–60 cm soil layers decreased by 35.57 and 75.53%, respectively. Similar to Cu, Zn also shows specific adsorption. It tends to form complexes with organic matter; thus, Zn does not easily migrate in soils and can easily become enriched, which affects its availability [12].

### 3.3. Interactions of Long-Term Application of Micronutrient Fertilizers on the Micronutrient Availability

#### 3.3.1. Interactions of Different Micronutrient Fertilizers on the Available Cu

**Figure 2:**
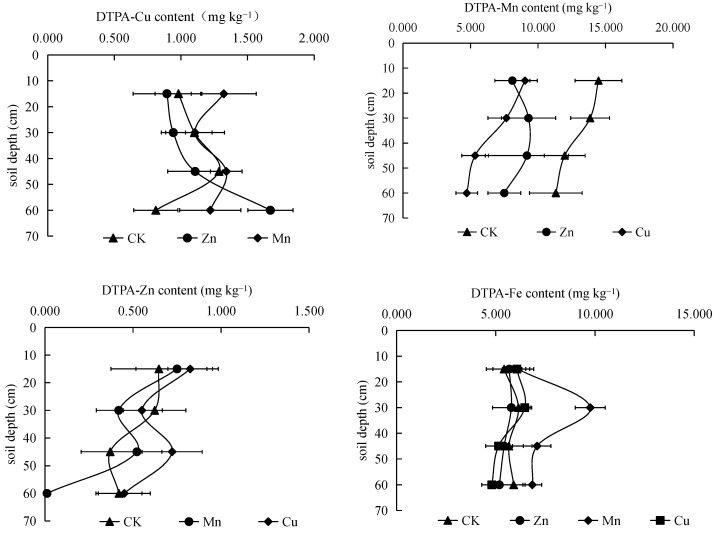
Interactions of long-term application of micronutrient fertilizers on the micronutrient availability. Values are means with standard deviations shown by the vertical bars (n = 3).

Zn and Mn fertilizers varied in their effects on the available Cu (Figure 2). The available Cu was lower in the 0–45 cm soil layer than the control, whereas it was higher in the 45–60 cm soil layer after 31 years of Zn application. Under the Zn application, the soil-available Cu increased with the soil depth. The Mn application increased the available Cu in all soil depths; specifically, the available Cu in the 0–15 and 45–60 cm soil layers were increased by 0.34 and 0.41 mg kg^−1^, respectively, compared with the control. Gilbey et al. demonstrated the need to strictly control the amount of Zn application in areas lacking in Cu and Zn, especially in sandy soils [26]. The excessive application of Zn might result in Zn-induced Cu deficiency in wheat and barley. Bowen et al. showed that Zn showed inhibitory effects on Cu absorption in plants, and the effect was even more evident under hydroponic culture conditions [27,28]. Chen et al. studied the effect of Zn and Mn applications on Cu nutrition in winter wheat; the results indicated that Zn fertilizer decreased the degree of Cu accumulation in wheat and negatively affected Cu absorption [29]. When low amounts of Mn were applied (0–39 mg kg^−1^), the amount of Cu accumulation in winter wheat increased as the amount of Mn applied increased, which showed that Mn application facilitated increases in soil-available Cu. However, when the amount of Mn applied was high (39–78 mg kg^−1^), the Cu accumulation in winter wheat decreased as the amount of Mn applied increased.

#### 3.3.2. Interactions of Different Micronutrient Fertilizers on the Available Mn

In the absence of fertilizer application, soil-available Mn decreased linearly with the soil depths (Figure 2). After the application of Zn and Cu, soil-available Mn also decreased linearly with the soil depths, but the decreases were all lower than the control. These results indicate that Zn and Cu fertilizers did not promote the accumulation of soil-available Mn. Chen et al. showed that there was an antagonistic relationship between Zn and Mn, which is consistent with the results of this study [29]. In addition to this, the results showed that the Cu fertilizer could not promote soil-available Mn. It has been described above that Mn fertilizer also did not significantly promote available Mn. Thus, we can conclude that the micronutrient fertilizers were not conducive to the increase in available Mn in the calcareous soils of the Loess Plateau.

#### 3.3.3. Interactions of Different Micronutrient Fertilizers on the Available Zn

The effects of micronutrient fertilizer application on soil-available Zn were similar in the control and experimental groups (Figure 2). The available Zn showed an S-curve pattern in the soil layers. The application of Cu and Mn increased soil-available Zn in the surface soil and 30–45 cm soil layer. These results indicate that Cu and Mn application did not consistently inhibit Zn in the soils. Millikan showed that the Zn concentration in subterranean clover and alfalfa was not affected when Cu was deficient [30]. This study also showed that Cu did not show inhibitory effects on Zn.

#### 3.3.4. Interactions of Different Micronutrient Fertilizers on the Available Fe

The micronutrient fertilizers varied in their effects on the soil-available Fe (Figure 2). In the absence of fertilizer application, the soil-available Fe in the different soil layers showed an S-curve pattern. The available Fe in the surface layer was lowest, followed by the 30–45 and 45–60 cm soil layers. The available Fe in the subsurface layer was the highest, reflecting the leaching and enrichment of Fe in the soil profile. In contrast to the control, Zn, Cu and Mn applications significantly increased the available Fe. The available Fe in the surface soils was increased by 0.26 mg kg^−1^ under the Zn fertilizer. The available Fe was increased by 0.64 and 0.32 mg kg^−1^ in the surface and subsurface layer, respectively, under the Cu fertilizer. Under Mn application, the Fe concentrations in the 0–15, 15–30, 30–45 and 45–60 cm soil layers were increased by 0.7, 3.62, 1.42, and 0.94 mg kg^−1^, respectively. The increase in soil-available Fe in the subsurface layer was the highest. Epstein and Stout showed that the amount of Fe absorption in crop roots increased when the Mn concentration in the clay suspension increased [31,32]; in addition, they showed that Mn could interfere with Fe transport from the roots to the branches. These findings could explain the increase in the available Fe in surface soils after Mn application. It might be associated with plant root absorption. The transport of elements from lower soils to upper soils through plant roots is one method by which plants can actively uptake nutrients. There are many hypothesized mechanisms underlying this mode of nutrient uptake, including carrier theory, the Donnan equilibrium and the potential gradient hypothesis (i.e., the “ion pump” hypothesis) [33]. Ion pumps are a group of special carrier proteins. They can drive reversible ATPases using external energy to promote the movement of all ions along their concentration gradient [34]. Somer et al. suggested that there is an antagonism between Fe and Mn [35]. Specifically, they showed that chlorotic plants in acidic soils contain a large amount of available Mn, which was not observed in our study. In this study, the Mn application promoted increases in the soil-available Fe.

#### 3.3.5. Interactions among Cu, Mn, Zn and Fe

The interactions among elements include antagonism and synergism. From the perspective of soil fertility, these refer to the interaction among elements in terms of availability. Through a certain transformation link, the solubility of each other can be increased or decreased, or the absorption of plants can be mutually promoted or inhibited through a certain mechanism [36]. There are also many examples of the excessive application of one element leading to the deficiency of another element, such as copper and zinc, copper and iron, manganese and iron, phosphorus and copper, phosphorus and iron, phosphorus and zinc, etc. [37,38]. This kind of phenomenon mostly occurs in plants and only partially in the soil. As for synergism, it is possible for various nutrient elements to exist within the normal supply level [12]. The long-term micronutrient fertilizer experiment provides a new research perspective for the interactions among soil Cu, Mn, Zn and Fe.

**Figure 3:**
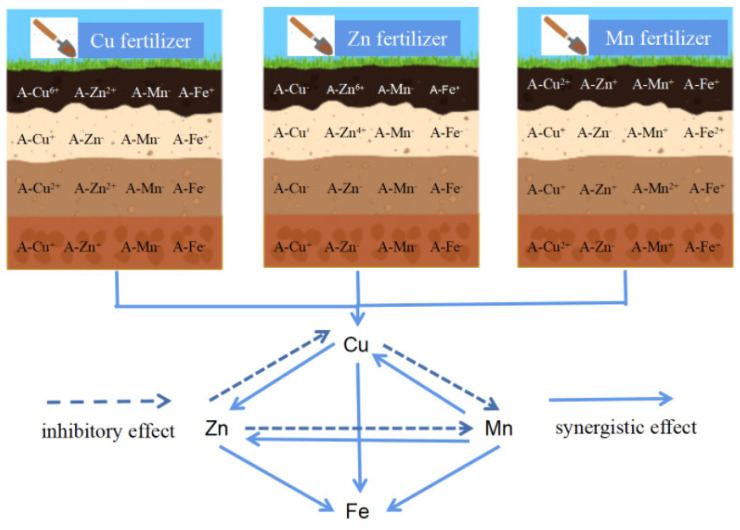
Interactions among Cu, Mn, Zn and Fe. A- represents the available state of micronutrients; + represents the increase in the available micronutrient; —represents the decrease in the available micronutrients; the numbers 6+, 2+ and 4+ represent the increase in the degree of the available micronutrients—the larger the number, the higher the increase.

Through the above analysis, the interaction among the micronutrients can be concluded. The picture (Figure 3) visually shows the changes in the available micronutrients in each soil layer (0–60 cm, interval was 15 cm) under different micronutrient fertilizer applications. In the surface soil, after long-term micronutrient fertilizer application, Zn, Cu and Mn fertilizers all promote the available Fe and, specifically, the Mn fertilizer. The Mn fertilizer promotes the available Fe, Zn and Cu. Fe is a good receiver, whereas Mn is a good importer. The Cu fertilizer greatly increases the available Zn. However, the Zn fertilizer suppresses Cu and Mn availability. The Cu fertilizer suppresses Mn availability.

Compared with existing research, this study did not show that there was a two-way antagonistic relationship between the elements but a one-way antagonism, such as the inhibition of zinc on copper. The relationships of manganese and iron, and copper and iron were also different from the previous studies [12]. Different research angles and experimental designs will lead to different results. In any case, the purpose of this study was to understand the interaction among elements from the perspective of the micronutrient fertilizers.

The micronutrient fertilizers showed a great contribution to Zn, Cu and Fe availability in the surface soil, whereas they showed a weak influence on Mn availability in this study. Wei et al. reported the same conclusion in the N and P fertilizer treatments in a continuous wheat system, whereas there was a significant increase in the N, P and organic fertilizer treatments and the long-term planting of leguminous crops which could also lead to an increase in available Mn on the Loess Plateau [5]. This indicated that wheat, the main crop on the Loess Plateau, might be not conducive to available Mn accumulation in the study area, and crop-legume rotation and N, P and organic fertilizer treatments might be a choice for improving available Mn. The leguminous plants acidified the rhizosphere soil; moreover, the leguminous plants showed moderate Mn absorption, which could result in the transformation of other Mn fractions [39]. Thus, crop rotation, especially with legumes, could ameliorate the Mn deficiency in the calcareous soil of the Loess Plateau.

### 3.4. Relationships between Soil Properties and Micronutrient Availability

Path analysis is suitable for handling more complex variable relationships, so the direct path coefficient and indirect path coefficient between available micronutrients and soil properties were analyzed using SPSS software (Table 2, Table 3, Table 4 and Table 5).

#### 3.4.1. Path Analysis between Soil Properties and DTPA-Zn

The path analysis between the soil properties and DTPA-Zn (Table 2) shows that the fulvic acid and organic matter were positively correlated with DTPA-Zn. Through the highest indirect path coefficient (0.114, 0.042) between fulvic acid and organic matter, we found that organic matter might affect the availability of soil Zn mainly through fulvic acid, whereas the soil pH, humic acid and soil moisture were negatively correlated with DTPA-Zn. Available P and soil CaCO_3_ showed a negative direct influence on DTPA-Zn. This was because the soil-available P showed the highest negative influence on soil CaCO_3_. Therefore, we should pay special attention to the interaction of Ca and Zn in calcareous soil, which could indirectly lead to the inhibition of P and Zn. In addition, P and Zn were easy to form zinc phosphate precipitation. P can increase the adsorption of Zn on oxides and iron aluminum hydroxides in the soil and the adsorption of Zn on calcium carbonate [12,40], which might be the reason for the negative effect between the available P and Zn.

#### 3.4.2. Path Analysis between Soil Properties and DTPA-Mn

The path analysis between the soil properties and DTPA-Mn (Table 3) shows that soil CaCO_3_ and soil moisture were positively correlated with DTPA-Mn. The soil pH and humic acid showed a negative effect on DTPA-Mn because of their negative correlation coefficients and direct path coefficients. Organic matter showed the highest direct path coefficient on DTPA-Mn (−1.052), and available P showed the most significant negative direct effect on DTPA-Mn (−0.040), whereas they both showed a positive effect on DTPA-Mn. The previous studies showed that the application of micronutrient fertilizers could not promote the increase in soil-available Mn. The path analysis shows that this might be due to the negative direct correlation between organic matter, pH and DTPA-Mn. The high soil pH on the Loess Plateau was not conducive to the release of DTPA-Mn. The high content of organic matter might make the soil in the Loess Plateau loose and easy to be lost, which decreased the content of DTPA-Mn. As seen from the indirect path coefficients, the organic matter affected DTPA-Mn through available P; this might be the result of phosphate precipitation [12]. The path analysis shows that DTPA-Mn can be promoted by the promotion of soil moisture, the improvement of soil reduction conditions and fulvic acid. Wei et al. and Mandal et al. both found that organic matter can achieve an increase in available Mn [5,41]. In common, they all have a positive correlation; the difference is that organic matter showed a negative indirect influence on DTPA-Mn. At the same time, reducing substances showed a significant positive effect on DTPA-Mn. This showed that the interactive effect between the soil properties was greater than any single soil property effect on micronutrient availability.

#### 3.4.3. Path Analysis between Soil Properties and DTPA-Cu

The path analysis between the soil properties and DTPA-Cu (Table 4) shows that soil organic matter, available P and reducing substances were positively correlated with DTPA-Cu; other soil properties all showed negative effects on DTPA-Cu except for fulvic acid. Fulvic acid had a negative direct path coefficient, whereas it had a positive correlation coefficient on DTPA-Cu. The path analysis shows that this was caused by soil CaCO_3_ because there was a negative indirect path coefficient (−0.061) between fulvic acid and soil CaCO_3_; moreover, soil CaCO_3_ had the most significant direct path coefficient. The early research reported that Cu appeared to precipitate as a carbonate of hydroxides [42]. It is well-known that Cu^2+^ tends to form strong bonds with organic matter [43]. As seen from the path analysis, the specific adsorption of organic matter was achieved through the negative correlation and path coefficient of fulvic acid and humic acid on DTPA-Mn, whereas its positive influence was achieved through available P. Cu phosphates are unstable and will eventually dissolve [44]; this might be used to explain the significant correlation relationship between available P and DTPA-Cu.

#### 3.4.4. Path Analysis between Soil Properties and DTPA-Fe

The path analysis between soil properties and DTPA-Fe (Table 5) shows that soil CaCO_3_ and soil moisture were positively correlated with DTPA-Fe, and humic acid showed the most significant negative effect on DTPA-Fe. Verloo et al. found that in alkaline soil, iron was more stable with an increased amount of the complex of humic acid, which might cause the reduction in Fe availability [45]. The soil pH and organic matter both had negative direct path coefficients, whereas they showed positive correlation coefficients on DTPA-Fe. The path analysis shows that this was caused by soil CaCO_3_ and available P because there were negative indirect path coefficients (−0.207 and −0.264, respectively). “Lime induced plant chlorosis” often appeared in calcareous soil, whereas there was a significant positive effect between CaCO_3_ and DTPA-Fe in this study. The reason might be that the application of micronutrient fertilizers could promote the content of DTPA-Fe compared with chemical fertilizers.

## 4. Conclusions

In general, long-term micronutrient fertilizers can improve the surface soil properties on the Loess Plateau. However, the interactive effect between soil properties was greater than any single soil property effect on micronutrient availability. Under the effect of micronutrient fertilizers and the soil environment, the available Cu and Zn in surface soils were significantly increased, whereas the application of Mn did not significantly increase the soil-available Mn. We should look for new ways to improve soil-available Mn in calcareous soils on the Loess Plateau. A path analysis showed that DTPA-Mn can be promoted by the promotion of soil moisture, the improvement of soil reduction conditions and fulvic acid. Wheat was the main crop on the Loess Plateau; in order to increase the accumulation of available Mn, crop-legume rotation and N, P and organic fertilizer treatments might be a choice.

The interactions between micronutrient fertilizers and micronutrient availability showed that synergism was possible for various nutrient elements to exist within the normal supply level, whereas one-way antagonism partially occurred in the soil under long-term micronutrient fertilizers, such as the inhibition of zinc on copper, copper on manganese and zinc on manganese. These studies show that we should reasonably apply micronutrient fertilizers according to the availability of micronutrients and the nutritional needs of crops.

The path analysis shows that micronutrient fertilizers affected the available micronutrients mainly due to organic matter, soil CaCO_3_, soil moisture, available P, fulvic acid and reducing substances. In general, we found the importance of organic matter in calcareous soils; it cannot only directly affect the availability of micronutrients but also indirectly affect their availability through the indirect interaction with fulvic acid, reducing substances, available P and CaCO_3_.

The results of this study are helpful to understand the effect of micronutrient fertilizer application on soil properties and micronutrient availability. Furthermore, additional work is warranted, such as the dynamic change in soil properties and micronutrient availability in different years. Further research is needed on the change mechanisms involved in main soil properties and micronutrient availability through micro experimental research in order to achieve a better understanding of how to use these experimental results in the light of concrete circumstances and realize more productive and sustainable agriculture.

## Figures and Tables

**Table 1:** Soil properties after 31 years of continuous application of micronutrient fertilizers.

Treatment	Depth cm	pH	Organic Matter g kg^−1^	CaCO_3_ g kg^−1^	Available P mg kg^−1^	Humic Acid g kg^−1^	Fulvic Acid g kg^−1^	Total Amount of Reducing Substances cmol kg^−1^	Soil Moisture %
The control treatment	0–15	8.15 b	10.62 bcd	108.8 c	26.09 a	3.47 a	2.14 cde	0.97 abcd	13.78 h
	15–30	8.29 ab	12.57 ab	128.0 a	13.38 c	2.93 ab	2.09 de	1.15 ab	13.86 gh
	30–45	8.39 ab	9.68 cdef	80.0 d	2.80 e	2.47 c	1.31 g	0.88 abcd	14.23 fg
	45–60	8.40 ab	8.72 def	70.4 de	3.10 e	3.07 ab	1.48 g	0.79 bcd	14.91 de
Zn fertilizer	0–15	8.32 ab	11.10 bc	79.2 d	11.68 c	1.21 d	1.75 f	1.01 abcd	14.56 ef
	15–30	8.40 ab	11.55 abc	74.4 de	7.73 d	1.34 d	6.83 a	1.04 abcd	14.59 ef
	30–45	8.51 a	8.37 ef	55.2 fg	1.25 e	0.60 d	1.79 f	0.76 cd	14.17 fgh
	45–60	8.49 a	8.43 ef	49.6 g	0.30 e	0.82 d	2.13 cde	0.77 cd	14.81 de
Mn fertilizer	0–15	8.35 ab	12.46 ab	112 bc	13.14 c	0.77 d	1.91 ef	1.13 abc	16.38 a
	15–30	8.39 ab	10.61 bcd	121.6 ab	3.40 e	0.88 d	2.16 cde	0.97 abcd	15.16 cd
	30–45	8.41 ab	8.46 ef	64 ef	0.70 e	0.65 d	2.11 de	0.77 cd	15.69 b
	45–60	8.41 ab	9.55 cdef	44.4 g	1.20 e	0.77 d	2.30 bcd	0.87 abcd	15.51 bc
Cu fertilizer	0–15	8.23 ab	13.23 a	73.6 de	21.59 b	0.91 d	2.50 b	1.21 a	14.26 fg
	15–30	8.35 ab	10.59 bcd	75.2 d	14.52 c	1.05 d	2.42 bc	0.96 abcd	14.18 fgh
	30–45	8.44 ab	7.89 f	52 g	1.60 e	3.07 ab	0.63 h	0.72 d	14.27 fg
	45–60	8.47 ab	10.13 cde	48.8 g	1.00 e	3.24 ab	0.28 i	0.92 abcd	14.95 de

Note: Means were compared using the Duncan’s multiple range test. Values with the same letter were not significantly different at *p* < 0.05.

**Table 2:** Path analysis between soil properties and DTPA-Zn.

Soil Properties	Direct Path Coefficient	Indirect Path Coefficient								*r*
		pH	Or	Ca	Av	Hu	Fu	Re	Mo	
pH	−0.178	-	−0.049	0.045	0.038	−0.001	0.000	0.016	−0.026	−0.154
Or	0.139	0.062	-	−0.077	−0.047	−0.004	0.114	0.088	−0.002	0.274 *
Ca	−0.131	0.061	0.082	-	−0.038	0.002	0.046	0.052	0.006	0.079
Av	−0.070	0.098	0.093	−0.071	-	0.003	0.075	0.057	0.029	0.214
Hu	0.024	0.004	−0.023	−0.008	−0.009	-	−0.115	−0.009	0.032	−0.104
Fu	0.379 *	0.000	0.042	−0.016	−0.014	−0.007	-	0.030	0.001	0.414 **
Re	0.107	−0.026	0.114	−0.063	−0.037	−0.002	0.105	-	−0.009	0.188
Mo	−0.082	−0.055	0.003	0.009	0.025	−0.009	−0.004	0.012	-	−0.102

Note: Or = organic matter; Ca = CaCO_3_; Av = available P; Hu = humic acid; Fu = fulvic acid; Re = reducing substances; Mo = soil moisture. *r* was the correlation coefficient between micronutrients and soil properties. * Values were significant at *p* < 0.05. ** Values were significant at *p* < 0.01. The letters and symbols in the following Table 3, Table 4 and Table 5 are the same as this table.

**Table 3:** Path analysis between soil properties and DTPA-Mn.

Soil Properties	Direct Path Coefficient	Indirect Path Coefficient								*r*
		pH	Or	Ca	Av	Hu	Fu	Re	Mo	
pH	−0.571 *	-	0.368	−0.248	0.022	0.002	0.000	0.109	0.132	−0.185
Or	−1.052	0.200	-	0.423	−0.027	0.014	0.044	0.615	0.009	0.224
Ca	0.720 **	0.197	−0.617	-	−0.022	−0.005	0.018	0.363	−0.030	0.622 **
Av	−0.040 **	0.313	−0.703	0.388	-	−0.010	0.029	0.401	−0.149	0.229
Hu	−0.082	0.014	0.173	0.046	−0.005	-	−0.044	−0.065	−0.165	−0.127
Fu	0.146	0.000	−0.317	0.086	−0.008	0.025	0-	0.208	−0.005	0.135
Re	0.751 *	−0.083	−0.861	0.348	−0.021	0.007	0.040	-	0.047	0.228
Mo	0.425 **	−0.178	−0.021	−0.051	0.014	0.032	−0.002	0.083	-	0.302 *

**Table 4:** Path analysis between soil properties and DTPA-Cu.

Soil Properties	Direct Path Coefficient	Indirect Path Coefficient								*r*
		pH	Or	Ca	Av	Hu	Fu	Re	Mo	
pH	−0.098	-	−0.072	0.174	−0.209	0.008	0.000	0.034	−0.046	−0.210
Or	0.207	0.034	-	−0.296	0.255	0.051	−0.056	0.190	−0.003	0.381 **
Ca	−0.505 **	0.034	0.121	-	0.206	−0.020	−0.022	0.112	0.010	−0.064
Av	0.382	0.054	0.138	−0.272	-	−0.038	−0.037	0.124	0.052	0.401 **
Hu	−0.310 *	0.002	−0.034	−0.032	0.047	-	0.057	−0.020	0.057	−0.233
Fu	−0.187	0.000	0.062	−0.061	0.076	0.094	-	0.064	0.002	0.050
Re	0.232	−0.014	0.169	−0.244	0.204	0.027	−0.052	-	−0.016	0.305 *
Mo	−0.147	−0.031	0.004	0.036	−0.134	0.120	0.002	0.026	-	−0.124

**Table 5:** Path analysis between soil properties and DTPA-Fe.

Soil Properties	Direct Path Coefficient	Indirect Path Coefficient								*r*
		pH	Or	Ca	Av	Hu	Fu	Re	Mo	
pH	−0.471	-	0.319	−0.207	0.217	0.008	0.000	0.123	0.053	0.042
Or	−0.911 *	0.165	-	0.352	−0.264	0.055	0.027	0.693	0.003	0.122
Ca	0.600 **	0.162	−0.535	-	−0.213	−0.022	0.011	0.410	−0.012	0.402 **
Av	−0.395	0.258	−0.609	0.324	-	−0.041	0.018	0.452	−0.060	−0.053
Hu	−0.336 *	0.012	0.150	0.039	−0.049	-	−0.027	−0.073	−0.066	−0.350 **
Fu	0.089	0.000	−0.275	0.072	−0.078	0.102	-	0.234	−0.002	0.143
Re	0.847	−0.069	−0.746	0.290	−0.211	0.029	0.025	-	0.019	0.185
Mo	0.170	−0.147	−0.019	−0.043	0.138	0.130	−0.001	0.093	-	0.322 *

## Data Availability

The data that support the findings of this study are available on request from the corresponding author.
